# *Agrobacterium*-mediated transient transformation of *Flaveria bidentis* leaves: a novel method to examine the evolution of C_4_ photosynthesis

**DOI:** 10.1186/s13007-024-01306-z

**Published:** 2024-12-27

**Authors:** Christopher J. Baros, Jeremy Beerkens, Martha Ludwig

**Affiliations:** School of Molecular Sciences, University of Western Australia, Crawley, WA 6009 Australia

**Keywords:** C_4_ photosynthesis, Evolution of C_4_ photosynthesis, *Flaveria*, Phospho*enol*pyruvate carboxylase, Transient transformation

## Abstract

The genus *Flaveria* has been studied extensively as a model for the evolution of C_4_ photosynthesis. Thus far, molecular analyses in this genus have been limited due to a dearth of genomic information and the lack of a rapid and efficient transformation protocol. Since their development, *Agrobacterium*-mediated transient transformation protocols have been instrumental in understanding many biological processes in a range of plant species. However, this technique has not been applied to the genus *Flaveria*. Here, an efficient protocol for the *Agrobacterium*-mediated transient transformation of the leaves of the C_4_ species *Flaveria bidentis* is presented. This technique has the distinct advantages of rapid turnaround, the ability to co-transform with multiple constructs, and the capacity to assay coding and non-coding regions of *Flaveria* genomes in a homologous context. To illustrate the utility of this protocol, the quantitative transcriptional regulation of phospho*enol*pyruvate carboxylase, the primary carboxylase of C_4_ plants, was investigated. A 24 bp region in the *ppcA1* proximal promoter was found to elicit high levels of reporter gene expression. The *Agrobacterium*-mediated transient transformation of *F. bidentis* leaves will accelerate the understanding of the biology and evolution of C_4_ photosynthesis in the genus *Flaveria* as well as in other C_4_ lineages.

**Supplementary Information:**

The online version contains supplementary material available at 10.1186/s13007-024-01306-z.

## Background

Given its high degree of convergence and propensity to confer tolerance in hot and dry conditions, the C_4_ phenotype is both conceptually remarkable and of great agronomic importance [1–5]. These characteristics have motivated the study of a plethora of C_4_ species, both monocotyledonous and eudicotyledonous, from a range of genera in many families [4]. However, among these C_4_ genera, the number, efficacy, and robustness of molecular transformation protocols are limited. Consequently, the perturbation and complementation of C_4_ syndromes at the genetic level through transformation technologies are restricted to relatively few species [6, 7].

From an evolutionary perspective, the genus *Flaveria* is attractive as it contains individual species that employ either a C_3_, C_3_-C_4_ intermediate, C_4_-like, or C_4_ photosynthetic pathway [8–11]. This continuum of photosynthetic evolution, from an ancestral C_3_ state to a derived C_4_ syndrome, was first determined from these early biochemical and physiological studies and has since been complemented by investigations into detailed leaf anatomy and phylogenetic, metabolomic, and transcriptomic analyses [12–17]. Chitty et al. [18] developed a stable *Agrobacterium*-mediated transformation protocol for the C_4_ species *F. bidentis*. This protocol is predicated on callus co-cultivation and plant regeneration, and has been used in numerous studies, including the interrogation of the C_4_ cycle and investigation of the transcriptional regulation of C_4_-associated genes using promoter-reporter constructs [19–32]*.* Consequently, it is from this protocol that the understanding of the molecular evolution of C_4_ photosynthesis in *Flaveria* has progressed immeasurably. However, obtaining a transgenic *F. bidentis* plant takes 15–20 weeks after inoculation of calli with *Agrobacterium* [18]. This is in contrast to the model organism *Arabidopsis thaliana*, for which transgenic plants can be assayed between four and six weeks after *Agrobacterium* inoculation [33]. Furthermore, the comparatively long life cycle of *F. bidentis* extends the time needed to generate homozygous lines [8, 34]. In addition to lacking expediency, the stable transformation *F. bidentis* requires specialty equipment, expensive consumables, and highly skilled practitioners [18]. Collectively, these characteristics make the molecular transformation of *F. bidentis* both temporally and logistically restrictive.

The aim of this study was to investigate whether the above limitations of the stable *F. bidentis* transformation system could be circumvented for some C_4_ evolutionary studies by the development and implementation of a transient transformation system. Unlike stable approaches, transient transformation does not involve the integration of DNA into the plant genome; instead, the DNA that enters the cell is expressed ephemerally [7, 35–38]. The advantages of transient approaches are the ability to rapidly generate experimental results—often within a working week [39], the ability to analyze genes that have deleterious effects on growth and development [39, 40], and the avoidance of somaclonal variation [41]. These advantages have motivated the development of transient transformation techniques in countless plant species, which have been extensively used in the characterization of gene function [42].

The use of *Agrobacterium* is common among plant transient transformation techniques. One variation of this technique, referred to as ‘agroinfiltration’, involves the syringe infiltration of *Agrobacterium* suspensions, which contain trangenes of interest, into the leaf parenchyma. This results in the accumulation of extrachromosomal transfer DNA (T-DNA) copies from which transgene expression can be detected, typically, 2–4 days post infiltration [35, 36, 38, 43, 44]. This method has become invaluable in plant molecular biology studies, providing a wealth of information into transcriptional dynamics, protein function, protein localization, and more [39, 45]. The most widely used agroinfiltration-based transient transformation protocol is for the Australian tobacco species, *Nicotiana benthamiana* [43]. Agroinfiltration protocols have been developed for other well-studied C_3_ plant species, such as *A. thaliana*, *Helianthus annuus*, and *Glycine max* [41, 46–48]. However, the use of these heterologous expression systems to study *Flaveria* genetic elements has been limited to highly ancestral processes, as outside of their endogenous context, insight into the *in planta* function of C_4_ transgenes is inadequate [29]. Consequently, existing heterologous expression systems are limiting for the characterization of *Flaveria* genetic elements that pertain to the evolution of C_4_ photosynthesis. This inability to assess and characterize *Flaveria* genetic elements in vivo and in a timely manner presents a severe bottleneck for the study of C_4_ photosynthesis.

Here, an *Agrobacterium*-mediated transient transformation protocol for *F. bidentis* leaves is reported. The system allows for the inexpensive, robust, and rapid testing of numerous *F. bidentis* genetic elements in a homologous context. This system will facilitate further study into the evolution of C_4_ photosynthesis in *Flaveria*, and C_4_ photosynthesis more broadly. To illustrate this, the newly developed *F. bidentis* leaf transient transformation system was used to investigate the quantitative regulation of the gene encoding the C_4_-associated phospho*enol*pyruvate carboxylase (*ppcA1*) from the C_4_ *Flaveria* species *F. trinervia*. Phospho*enol*pyruvate carboxylase (PEPC) is the primary carboxylase of C_4_ plants and its exaptation into the C_4_ pathway in the genus *Flaveria* is dependent upon quantitative and qualitative changes to the expression of its cognate gene [49]. The qualitative changes in *ppcA1* expression have been attributed to action of the mesophyll expression module 1 (MEM1) element, which has been shown to confer mesophyll cell-specific expression to downstream genes [22]. This element is present in the upstream regions of C_4_ *Flaveria ppcA1* genes and absent from the orthologous regions of C_3_ *Flaveria* species [22, 50]. At the quantitative level, an elevated abundance of *ppcA1* transcripts is observed in C_4_ *Flaveria* species, relative to that of the orthologs of their C_3_ congeners [51]. However, the *cis*-regulatory elements (CREs) – if any – responsible for this discrepancy in quantitative expression are as yet unknown.

Experiments using stably transformed *F. bidentis* plants have shown that although the MEM1 element is sufficient for, and indispensable to, the mesophyll cell-specific expression of C_4_ *Flaveria ppcA1*, a high degree of mesophyll cell-specific expression is contingent on both the presence of the MEM1 element *and* the *F. trinervia ppcA1* proximal promoter [22, 24]. The *F. trinervia ppcA1* proximal promoter has been designated as the 570 bp upstream of the translational start site of the protein encoded by *ppcA1* [22, 24]. Consequently, it has been proposed that there is a cooperative relationship between the MEM1 enhancer and a CRE (or CREs) harbored in this proximal region [22, 24]. Previous work has predicted putative transcription factor binding sites and CREs throughout this region; however, no element responsible for the high level of expression has been experimentally determined [24, 52]. In this study, the *F. trinervia ppcA1* proximal promoter was interrogated using promoter-reporter constructs to illustrate the efficacy and applicability of the newly developed *F. bidentis* leaf transient transformation system to study the evolution of this key C_4_-associated enzyme. In doing so, a 24 bp region in the *F. trinervia ppcA1* proximal promoter was identified that is responsible for a high level of reporter gene expression.

## Methods

### Plant propagation and growth

*Flaveria bidentis* (L.) Kuntze plants were grown in a growth cabinet at 25 ºC under a 12 h photoperiod using cool-white panel lights (45 µmol m^−2^ s^−1^). Plants were grown in pots of 64 mm diameter × 60 mm height, with a substrate prepared at a 6:1:1 ratio of soil:perlite:vermiculite. *F. bidentis* plants were either propagated from seed or via cuttings using Rootex-G (Fernland; according to the manufacturer’s instructions). Leaves suitable for transformation were present on seed-grown plants ~ 8 weeks after sowing, whereas plants grown from cuttings exhibited transformable leaves ~ 4 weeks after propagation.

### *Agrobacterium* competent cell preparation and transformation

*Agrobacterium tumefaciens* (GV3101) was used to inoculate 3 mL of Luria–Bertani broth (LB) supplemented with 50 μg mL^−1^ rifampicin and incubated overnight at 28 °C with shaking at 180 rpm. This culture was then used to inoculate 50 mL of LB containing 50 μg mL^−1^ rifampicin and this subculture was incubated overnight at 28 °C with shaking at 200 rpm until an OD_600_ of 0.75 was reached. After chilling on ice for 10 min, cells were collected at 3,000 x*g* for 10 min at 4 °C, then washed once with, and resuspended in, 1 mL of ice-cold 20 mM CaCl_2_. These chemically competent cells were then aliquoted into microfuge tubes, snap frozen in liquid nitrogen, and stored at −80 °C until use.

Chemically competent *Agrobacterium* cells were transformed using a modified freeze–thaw method [53], except LB was used in the recovery step. Glycerol stocks were prepared from *Agrobacterium* cultures harboring binary plasmids and stored at −80 °C.

### *Agrobacterium* suspension preparation and canonical leaf infiltration conditions

Once thawed, *Agrobacterium* glycerol stocks were used to inoculate LB containing 50 μg mL^−1^ rifampicin and 50 μg mL^−1^ kanamycin. Cultures were incubated for 24 h at 28 °C with shaking at 200 rpm until an OD_600_ of 1.0–2.0 was reached.

For experiments that did not include co-transformation with the vector containing the sequence encoding *Cymbidium ringspot virus* p19 protein (*p19* vector), *Agrobacterium* cells were collected at room temperature at 2,000 *xg* for 10 min and resuspended in freshly prepared leaf infiltration buffer (10 mM 2-(*N*-morpholino)ethanesulfonic acid, pH 5.6, 10 mM MgCl_2_, 1 mM acetosyringone) to a final OD_600_ of 1.0. For these experiments, leaf infiltration buffer without *Agrobacterium* served as a negative control.

For experiments that included co-transformation with the *p19* vector, *Agrobacterium* cells were collected as above, and resuspended in leaf infiltration buffer to a final OD_600_ of 1.3 (experimental culture OD_600_ = 1.0; *p19* vector culture OD_600_ = 0.3). For these experiments, the negative control consisted of the *p19* vector culture resuspended in leaf infiltration buffer to an OD_600_ of 0.3.

*Agrobacterium* suspensions were incubated at room temperature for 2 h with gentle inversion every 30 min. Before infiltration, 0.01% [v/v] Silwet L-77 (PhytoTech Labs) was added to each suspension. *Agrobacterium* suspensions were infiltrated into the *F. bidentis* leaf tissue on the abaxial surface using a 1 mL plastic syringe.

### Optimization of leaf zone and acetosyringone concentration

For experiments investigating the optimal leaf zone for transient transformation efficiency, a co-transformation infiltration suspension was prepared in which leaf infiltration buffer contained one *Agrobacterium* strain that carried a vector with a gene encoding the green fluorescent protein (GFP) and another strain harboring the *p19* vector (see above). Genes encoding both GFP and p19 were under the control of the cauliflower mosaic virus 35S (CaMV) promoter. The negative control for these experiments consisted of leaf infiltration buffer containing only the *Agrobacterium* strain harboring the *p19* vector. These *Agrobacterium* suspensions were infiltrated along the length of the *F. bidentis* leaf blade. Three days post infiltration, 10 mm diameter leaf disks were excised from three leaf zones, ~ 20 mm, ~ 60 mm, and ~ 80 mm, from the petiole; referred to as the proximal, upper-middle, and distal leaf zones, respectively. Transformation efficiency was calculated as described below.

To optimize acetosyringone concentration, the co-transformation infiltration suspension containing the *Agrobacterium* strains harboring the GFP and *p19* constructs described above was prepared with three different acetosyringone concentrations: 0.1 mM, 0.5 mM, or 1.0 mM. The negative controls for these experiments consisted of leaf infiltration buffers containing the different acetosyringone concentrations and the *Agrobacterium* strain harboring the *p19* vector. Leaves were infiltrated in the upper-middle zone, leaf disks were harvested 3 days post infiltration, and transformation efficiency was calculated (see below).

### Efficiency of *Flaveria bidentis* leaf transient transformation

Nine *F. bidentis* leaves were transiently transformed with the co-transformation infiltration suspension described above containing 1 mM acetosyringone on three separate occasions. Three days after infiltration, leaf disks were harvested from the upper-middle zone of each leaf and transformation efficiency was calculated (described below). The overall transformation efficiency was calculated based on the mean transformation efficiencies of these nine leaves. Leaf infiltration buffer containing only the *Agrobacterium* strain harboring the *p19* vector served as the negative control for these experiments.

Within each leaf disk, three random sets of brightfield and corresponding GFP epifluorescence images (excitation: 454–490 nm; emission: 500–540 nm; laser power: 50%; gain: 1.0X) were captured using a Nikon Ti2 inverted microscope at 40X magnification. The transformation efficiency was calculated from each image by dividing the total number of GFP expressing cells by the total number of cells. Only cells in the plane of focus were included in these analyses. This value constituted the transformation efficiency of a leaf disk. Each quantification assay was repeated three times per transformation experiment.

### Transformability of *Flaveria bidentis* leaf cell-types

To investigate the transformability of different cell types within the *F. bidentis* leaf, leaves were infiltrated with an *Agrobacterium* suspension harboring a gene coding for GFP under the control of the *F. bidentis* carbonic anhydrase 3 promoter region (Fbca3) [29], and an *Agrobacterium* suspension harboring a GFP reporter gene fused to the 5′- and 3′-untranslated regions of the *F. bidentis* ribulose-1,5-bisphosphate carboxylase/oxygenase small subunit 1 (*FbRbc*S1) [54] under the control of the CaMV promoter. Leaf infiltration buffer containing no *Agrobacterium* was used as the negative control for these experiments. Leaves were visualized 3 days post-infiltration.

### Generation of promoter-reporter constructs

Using *F. trinervia* genomic DNA as a template, a 2184 bp fragment containing the distal and proximal regions of the *ppcA1* promoter [22], was amplified with primers incorporating *Xba*I and *Nco*I restriction endonuclease sites at the 5′- and 3′-ends, respectively (*FtppcA1* upstream region FWD and REV primers; Supplementary Table S1). This fragment was inserted into the pGEM-T Easy vector (Promega) as per the manufacturer’s instructions and its sequence was determined to confirm its identity. To generate truncated promoter-reporter constructs, fragments of differing lengths (570, 543, 472, and 362 bp) were amplified using forward primers designed against the *F. trinervia ppcA1* proximal promoter [22] and containing a flanking *Xba*I site (Supplementary Table S1) and the aforementioned REV primer, with the 2184 bp *F. trinervia ppcA1* promoter construct as a template. These amplicons were inserted into pGEM-T Easy (Promega) as described above, and their identities were confirmed via sequence determination. Following sequence confirmation, promoter fragments were excised by *Xba*I/*Nco*I double digest and inserted into the pMDC99-AK156 vector backbone digested with the same restriction endonucleases. The pMDC99-AK156 vector is a binary vector containing a firefly and *Renilla* luciferase expression cassette [55, 56]. In the construct used in this study, the *Renilla* luciferase transgene (*Rluc*) is under the constitutive control of the *Arabidopsis thaliana TCTP1* promoter, enabling luminescence normalization and accounting for variations in transformation efficiency, whereas the firefly luciferase gene (*Fluc*) is under the control of the experimental upstream regions. The resultant vectors were designated *FtppcA1(–570)::LUC*, *FtppcA1(–543)::LUC*, *FtppcA1(–472)::LUC*, and *FtppcA1(–362)::LUC*. The sequences of the plasmids were determined before they were introduced into *Agrobacterium tumefaciens* (GV3101) cells.

A second set of truncated promoter constructs was generated by synthesizing three additional *F. trinervia ppcA1* promoter fragments (Twist Bioscience). Three deletion constructs were generated to interrogate four regions, each between 24 and 35 bp in length. The three fragments began at positions −448, −421, and −396 relative to the PPCA1 translational start site. The 5′-ends of the fragments contained an *Xba*I and the 3′-ends contained a modified translational start site encompassed in an *Nco*I site, as described above. The promoter fragments were then digested from the pTWIST high copy number vector (Twist Bioscience) and inserted into the pMDC99-AK156 backbone, as described above, and designated *FtppcA(*−*448)::LUC*, *FtppcA(*−*421)::LUC*, and *FtppcA(*−*396)::LUC*.

The *F. pringlei ppcA* proximal promoter region was amplified from *F. pringlei* genomic DNA using primers incorporating *Xba*I and *Nco*I restriction endonuclease sites at the 5′- and 3′-ends, respectively (*FpppcA* upstream region FWD and REV primers; Supplementary Table S1). This fragment was inserted into the pGEM-T Easy vector (Promega) as per the manufacturer’s instructions and its sequence was determined to confirm its identity. The proximal promoter fragment was inserted into the pMDC99-AK156 vector backbone as described above, and was designated *FpppcA(*−*617)::LUC*. The sequence of this plasmid was determined before it was introduced into *Agrobacterium tumefaciens* (GV3101) cells.

### Luciferase assays

For this work, a transformation event was designated as the transient transformation of two *F. bidentis* leaves, each belonging to different, individual, non-clonal plants. For complete datasets of each construct, transformation events were repeated three times with each repetition spaced at intervals of greater than one week. Each transformation event also included a no-*Agrobacterium* control, for which two *F. bidentis* leaves were infiltrated with only leaf infiltration buffer, and two leaves transformed with the *F. trinervia ppcA1* proximal promoter, *FtppcA(*−*570)::LUC,* for background subtraction and normalization. Two leaf disks were harvested per transformed leaf; consequently, a total of at least eight leaf disks were harvested and included in the analyses for each construct.

Leaf disks were excised from the infiltrated areas of *F. bidentis* leaves using a 10 mm diameter tissue punch. The soluble protein was extracted from each leaf disk using 500 μL of protein extraction buffer (50 mM Tris, pH 7.5, 150 mM NaCl, 10% [v/v] glycerol, 0.1% [v/v] Tween-20, 1 mM phenylmethylsulfonyl fluoride, 1 mM dithiothreitol (DTT), 1X cOmplete Protease Inhibitor Cocktail (Roche)) on ice using a ground glass homogeniser.

Dual-luciferase assays were performed in a 96-well black plate (Greiner), using 20 µL of protein extract per reaction in technical triplicates. The assays were performed at room temperature on a CLARIOstar Plus microplate reader (BMG Labtech) employing a custom protocol programmed for the use of two injectors. In each plate well, the assay was initiated by the injection of 100 µL of firefly luciferase assay buffer (25 mM glycylglycine, 15 mM K_2_PO_4_/KH_2_PO_4_, pH 8.0, 4 mM ethylene glycol-bis(*β*-aminoethyl ether)-N,N,N′,N′-tetraacetic acid (EGTA), 2 mM ATP, 1 mM DTT, 15 mM MgSO_4_, 0.1 mM Coenzyme A, 75 µM D-luciferin; final pH 8.8) at a speed of 300 μL s^−1^ [73]. Initiation was followed by 1 s of shaking at 500 rpm and luminescence was captured for 10 s. The firefly luciferase reaction was quenched, and *Renilla* luciferase reaction initiated, both via the injection of 100 µL of *Renilla* luciferase assay buffer (1.1 M NaCl, 2.2 mM Na_2_EDTA, 0.22 M K_2_PO_4_/KH_2_PO_4_, pH 7.8, 0.44 mg mL^−1^ bovine serum albumin, 1.3 mM NaN_3_, 1.43 µM coelenterazine; final pH 5.0) at a speed of 300 μL s^−1^ [57]. This was followed by 1 s of shaking at 500 rpm and the resultant *Renilla* luminescence was captured for 10 s.

### Statistical analyses

The mean firefly and *Renilla* luminescence readings obtained from the no-*Agrobacterium* infiltration control samples were subtracted from the luciferase readings of the experimental constructs. Samples with *Renilla* luciferase readings that did not exceed background readings by a factor of five were removed from subsequent statistical analyses. For all other samples, the luminescence ratio of the adjusted readings was calculated for each technical replicate, and the luminescence ratio of each leaf disk was calculated from the mean of these technical triplicates. Each luciferase assay plate was treated independently, and samples were normalized to the mean luciferase ratio of *FtppcA1(−570)::LUC* that had been infiltrated in the same transformation experiment. Unpaired t-tests were performed to determine significant differences between the constructs (*α* = 0.05).

## Results

### Optimization of parameters for transient gene expression in *Flaveria bidentis* leaves

**Fig. 1:**
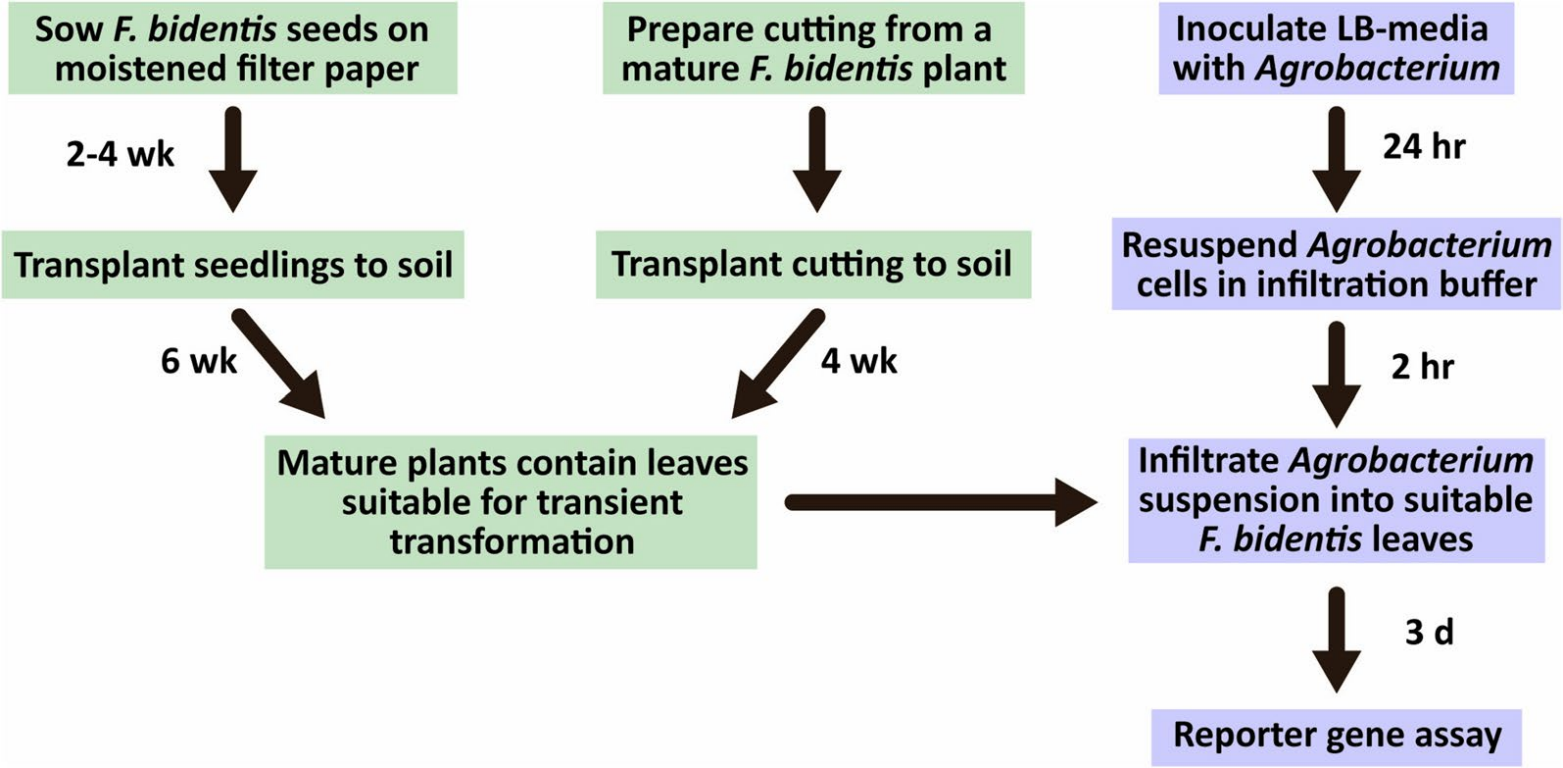
Schema of the *Agrobacterium*-mediated transient transformation of *Flaveria bidentis* leaves. Green boxes denote plant preparation steps, purple boxes denote *Agrobacterium* preparation and subsequent transformation steps. Arrows indicate direction of workflow with the duration between steps noted beside each arrow and represented in either hours (hr), days (d), or weeks (wk). LB, Luria–Bertani medium. See ‘Methods’ for details

The robust, replicable, and efficient protocol for the *Agrobacterium*-mediated transient transformation of *F. bidentis* leaves, from infiltration to analysis, takes 3 days (Fig. 1). Several parameters required optimization during the development of the protocol, including leaf selection, zone of infiltration, and acetosyringone concentration.

**Fig. 2:**
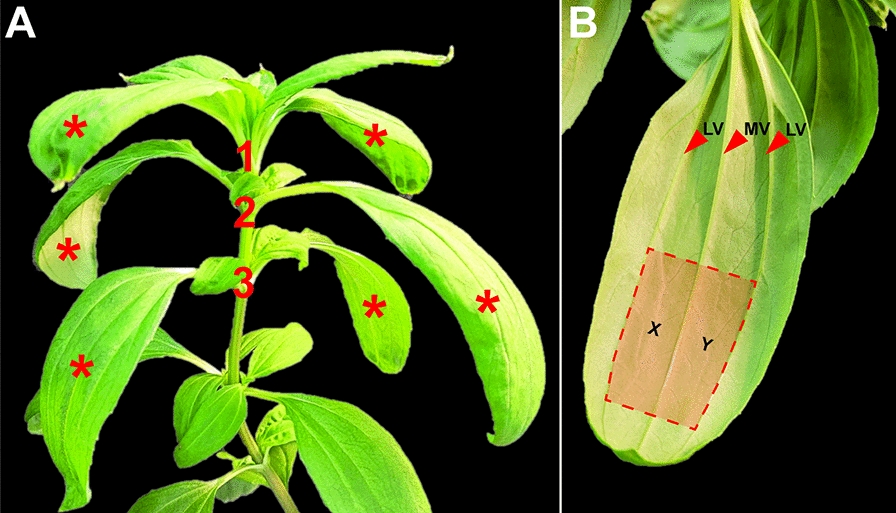
*Flaveria bidentis* leaves suitable for transient transformation. **A** A mature *Flaveria bidentis* plant with leaf pairs labelled at the nodes, starting at the first fully expanded leaf pair 1. Leaves labelled with a red asterisk are suitable for transient transformation. **B** *F. bidentis* leaf suitable for transformation viewed from the abaxial surface. The mid-vein (MV) and lateral veins (LV) are marked with red arrowheads. The leaf area that yields the greatest and most reproducible transformation efficiency is highlighted in red. This area is bounded by the LVs and bisected by the MV. Regions X and Y are marked in this area on either side of the MV. No discernible transfer of infiltrate across the MV has been observed; therefore, regions X and Y can be infiltrated with different *Agrobacterium* suspensions

Leaf selection is critical for transformation efficiency, reproducibility, and efficacy. Namely, considerations of leaf development, health, size, and region must be made. In addition, biological and technical replicates must be considered. For example, on a mature *F. bidentis* plant, the first pair of fully expanded leaves and the two leaf pairs below are usually suitable for transformation (Fig. 2A). Leaves showing no signs of damage, chlorosis, or senescence should be used. Furthermore, leaves should be large enough for ease of infiltration and subsequent analyses; typically, ~ 100 mm in length, with a leaf interveinal distance, from midvein (MV) to each lateral vein (LV), of ≥ 10 mm (Fig. 2B).

**Fig. 3:**
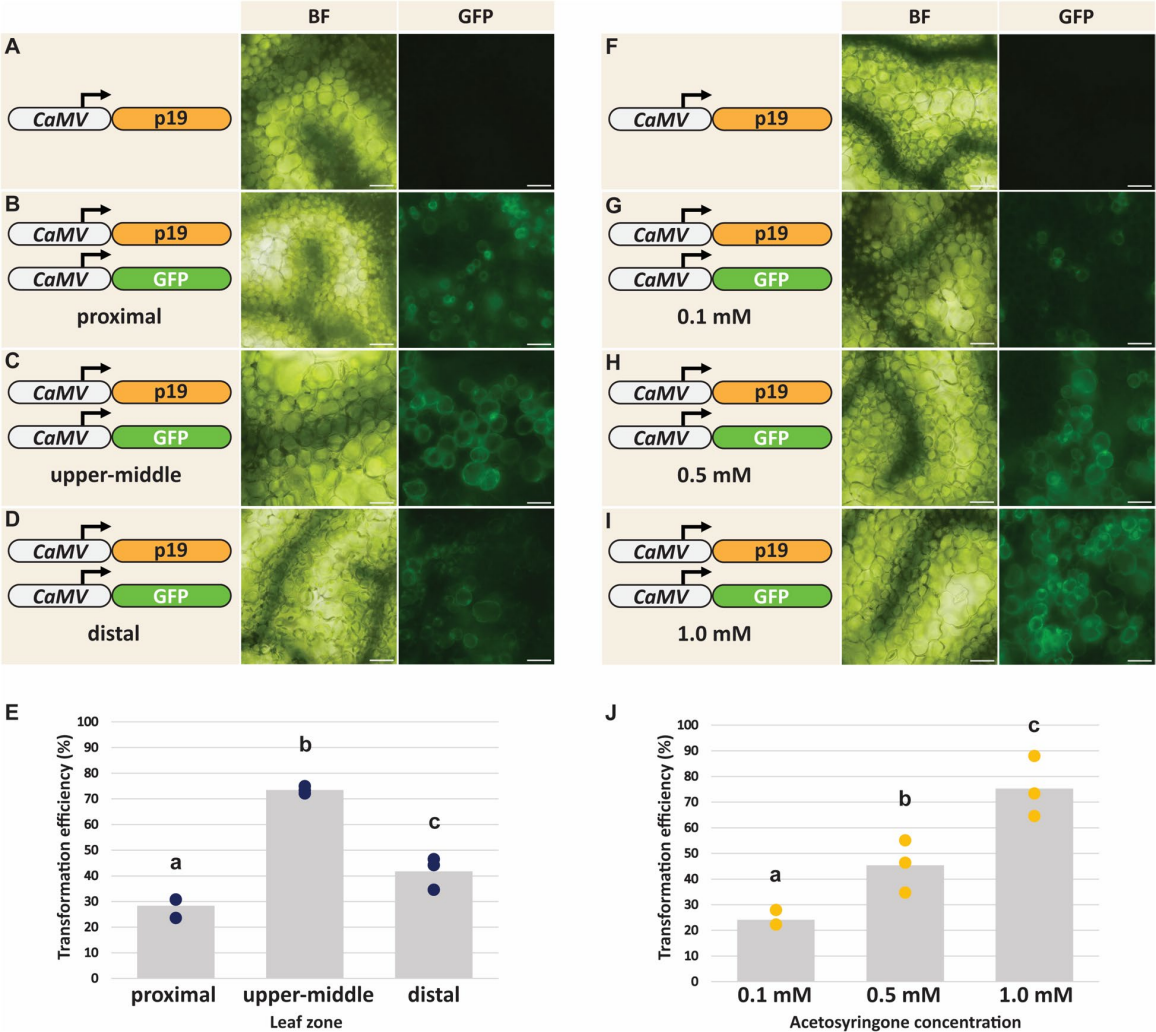
Optimization of the transformation efficiency of the *Agrobacterium*-mediated *Flaveria bidentis* leaf transient transformation protocol. **A** and **F** *Flaveria bidentis* leaves transformed with an *Agrobacterium* suspension harboring a vector containing a gene encoding p19 under the control of the cauliflower mosaic virus 35S (CaMV) promoter. **B**–**D** and **G**–**I** *F. bidentis* leaves transformed with an *Agrobacterium* co-suspension of one strain containing a gene encoding the green fluorescent protein (GFP) under the control of the CaMV promoter, and a strain harboring the p19 vector. **A**–**E** Efficiency of the *Agrobacterium*-mediated *F. bidentis* leaf transient transformation system at different leaf developmental zones; negative control using the upper-middle zone and representative of all three leaf zones (**A**), the proximal (**B**), upper-middle (**C**), and distal (**D**) leaf zones. (**E**) Quantification of transformation efficiency along the developmental gradient. Three leaves were transiently co-transformed. For each developmental zone, the number of transformed and untransformed cells in three fields of view were counted, and transformed cells were expressed as a percentage of total cells. Each data point (blue) represents the mean transformation efficiency from one developmental zone of a single leaf, as calculated from the mean of three fields of view. Columns represent the mean transformation efficiency of the developmental zone from three leaves. **F**–**J** Efficiency of the *Agrobacterium*-mediated *F. bidentis* leaf transient transformation system at different acetosyringone concentrations. Each *Agrobacterium* co-suspension contained a different concentration of acetosyringone in the leaf infiltration buffer; 0.1 mM (**G**), 0.5 mM (**H**), and 1.0 mM (**I**). An acetosyringone concentration of 1.0 mM was used in the transformation control leaf represented in (**F**). **J** Each data point (yellow) represents the mean transformation efficiency of a single leaf. Columns represent the mean transformation efficiency conferred by each acetosyringone concentration. For all experiments, leaf disks were excised 3 days post-infiltration and were visualized via brightfield (BF) and epifluorescence microscopy using a GFP filter set (GFP; excitation: 454–490 nm, emission collection: 500–540 nm). Scale bar = 30 μm

To determine the leaf developmental zone yielding the greatest transformation efficiency, *Agrobacterium* cells that harbored a binary plasmid containing a green fluorescent protein (GFP) reporter gene under the control of the CaMV promoter [58] and cells with a plasmid containing the sequence encoding the *Cymbidium ringspot virus* p19 protein also under the control of the CaMV promoter [44] were used. These two *Agrobacterium* populations were resuspended in the same leaf infiltration buffer containing 1 mM acetosyringone and infiltrated into the *F. bidentis* leaf lamina from the abaxial side (Fig. 2B; see Methods). Leaves were infiltrated along the length of the leaf blade (Figs. 2B and 3A–E). After 3 days, leaf disks were excised from three zones; ~ 20 mm, ~ 60 mm, and ~ 80 mm from the petiole; henceforth, referred to as the proximal, upper-middle, and distal leaf zones, respectively (Fig. 3A–E). Transformation efficiency was calculated as the percentage of cells expressing GFP, relative to the total number of cells in a field of view, as observed via epifluorescence and brightfield microscopy, respectively [48]. As shown in Fig. 3E, infiltration of the upper-middle leaf zone resulted in the greatest transformation efficiency (73.5%). The proximal zone yielded the lowest transformation efficiency (28.4%) of the three zones tested, whereas the transformation efficiency of the distal zone was 41.7%.

Acetosyringone is a phytohormone which is commonly used in transient transformation systems to induce *Agrobacterium* virulence and improve transient transformation efficiency [59, 60]. To investigate the concentration of acetosyringone required in the leaf infiltration buffer to yield a high transformation efficiency, *Agrobacterium* suspensions were incubated in leaf infiltration buffer containing 0.1 mM, 0.5 mM, or 1.0 mM acetosyringone for 2 h, before infiltration into the *F. bidentis* leaf parenchyma. Cells in the upper-middle leaf zone were used to calculate transformation efficiency, as described above. Among these three acetosyringone treatments, *Agrobacterium* suspensions incubated with 1.0 mM acetosyringone resulted in the greatest transformation efficiency (75.3%) and 0.1 mM acetosyringone exhibited the lowest transformation efficiency (24.1%) (Fig. 3F–J). Incubation with 0.5 mM acetosyringone exhibited a transformation efficiency intermediate to the 0.1 mM and 1.0 mM treatments (45.4%; Fig. 3F–J).

**Fig. 4:**
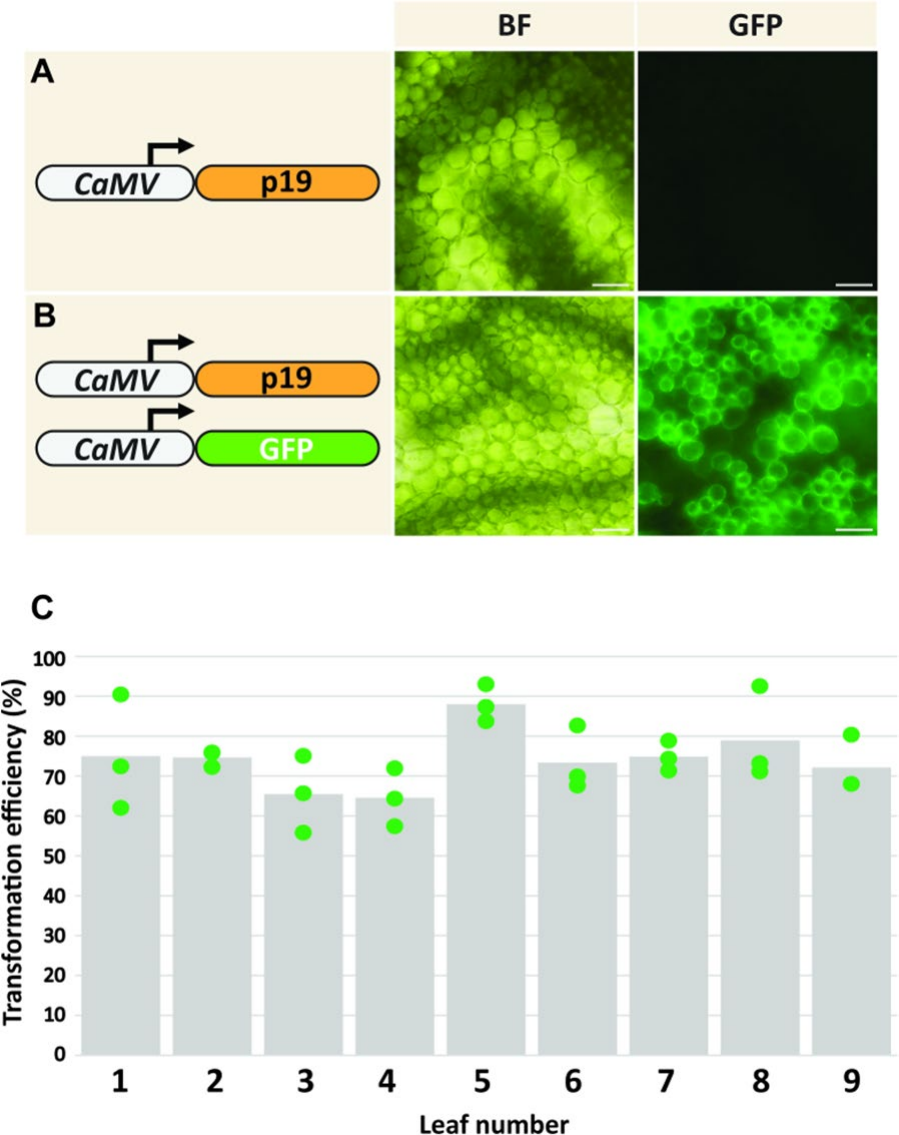
Efficiency of the *Agrobacterium*-mediated *Flaveria bidentis* leaf transient transformation system. **A** Leaf transformed with an *Agrobacterium* suspension harboring a vector containing a gene encoding p19 under the control of the cauliflower mosaic virus 35S (CaMV) promoter. **B** Leaf transformed with an *Agrobacterium* co-suspension of one strain containing a gene encoding the green fluorescent protein (GFP) under the control of the CaMV promoter, in addition to a strain harboring the p19 vector. Leaves were visualized 3 days post-infiltration via brightfield (BF) and epifluorescence microscopy with a GFP filter set (GFP; excitation: 454–490 nm, emission collection: 500–540 nm). Scale bar = 30 μm. **C** Quantification of transformation efficiency. Three leaves were transiently co-transformed on three different occasions (leaves 1–3, 4–6, and 7–9). The number of transformed and untransformed cells in three fields of view were counted and the transformed cells expressed as a percentage of the total. Each data point (green) represents the transformation efficiency in one field of view, and columns represent the mean transformation efficiency per leaf

As a result of the above optimization steps, a canonical protocol, with leaf infiltration buffer containing 1.0 mM acetosyringone and infiltrating the upper-middle leaf zone, was used to transiently transform a total of nine *F. bidentis* leaves across three independent experiments (Fig. 4). The mean transformation efficiency across these experiments was calculated to be 74.1% with a range of 64.5% to 88.0% (Fig. 4).

### Transformation of *Flaveria bidentis* leaf bundle-sheath cells

**Fig. 5:**
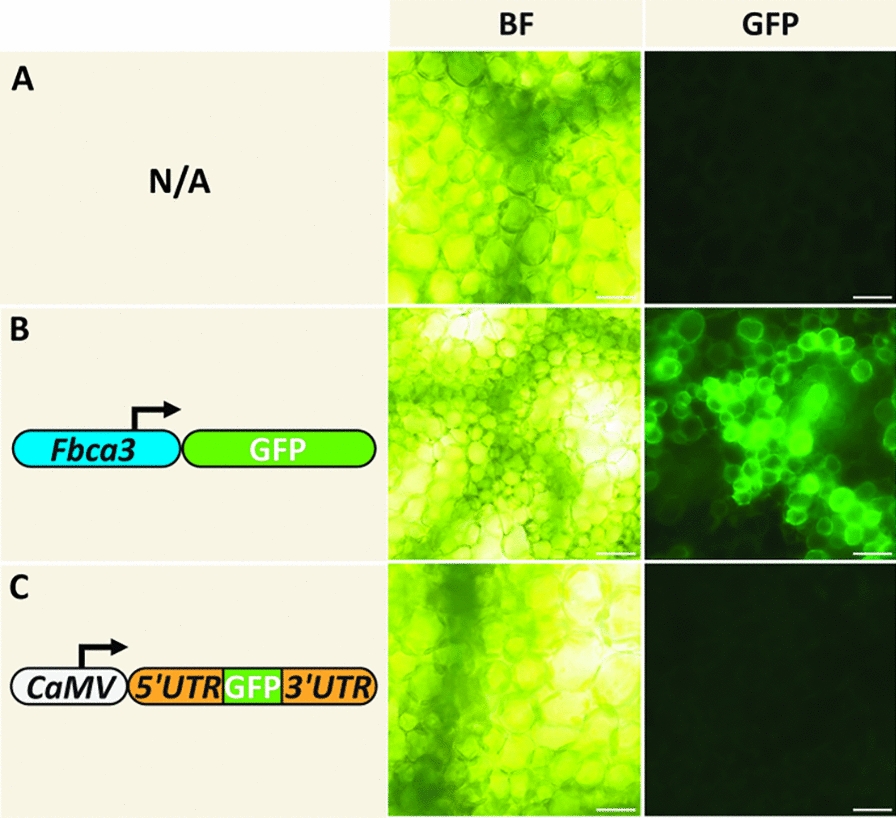
Transformability of *Flaveria bidentis* leaf cell types. **A** *Flaveria bidentis* leaf infiltrated with buffer containing no *Agrobacterium*. **B** *F. bidentis* leaf infiltrated with an *Agrobacterium* suspension harboring a gene encoding the green fluorescent protein (GFP) under the control of the upstream region of the gene encoding *F. bidentis β*-carbonic anhydrase 3 (*Fbca3*). **C** *F. bidentis* leaf infiltrated with an *Agrobacterium* suspension containing a gene encoding GFP fused to the 5′- and 3′-untranslated regions (UTR) of *F. bidentis* ribulose-1,5-bisphosphate carboxylase/oxygenase small subunit 1 and under the control of the cauliflower mosaic virus promoter (CaMV). Leaves were visualized 3 days post-infiltration via brightfield (BF) and epifluorescence microscopy with a GFP filter set (GFP; excitation: 454–490 nm, emission collection: 500–540 nm). Scale bar = 30 μm

To determine the transformability of leaf cell types, mesophyll cell-specific and bundle-sheath cell-specific *gfp* constructs were used to transiently transform *F. bidentis* leaves (Fig. 5). For mesophyll cell specificity, the upstream region of the gene encoding *F. bidentis β*-carbonic anhydrase 3 coupled to a *gfp* reporter gene was used (Fig. 5B). For bundle-sheath cell specificity, a *gfp* construct fused between the 5′- and 3′-untranslated regions of the *F. bidentis* ribulose-1,5-bisphosphate carboxylase/oxygenase small subunit 1 gene was used (Fig. 5C). The mesophyll cell-specific construct resulted in numerous transformed leaf mesophyll cells (Fig. 5B). By contrast, no transformed cells were visible in leaves infiltrated with the bundle-sheath cell-specific construct (Fig. 5C).

### Using the transient transformation method to quantify promoter strength

**Fig. 6:**
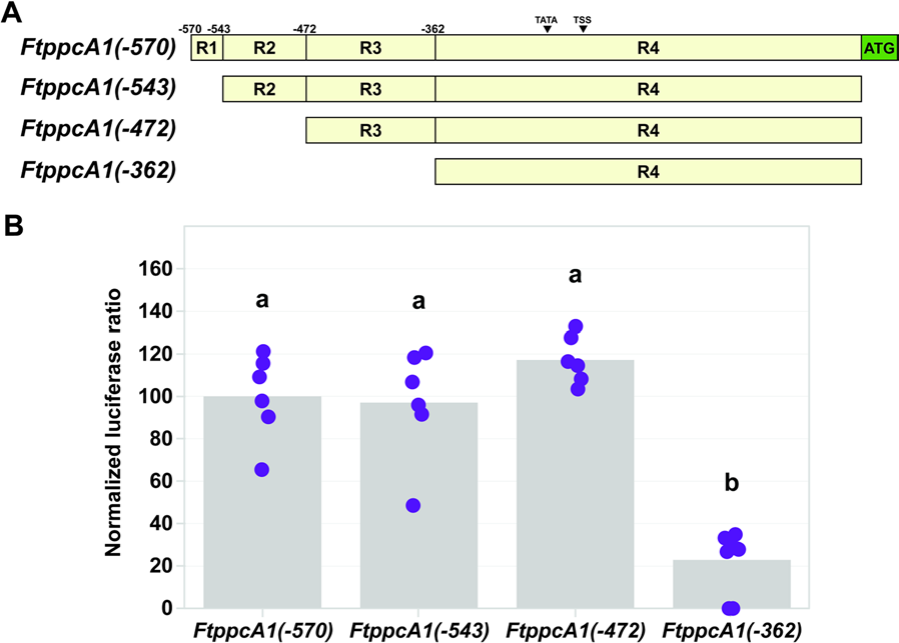
Quantification of promoter strength using *Flaveria bidentis* leaf transient transformation. **A** Maps of *Flaveria trinervia* C_4_-associated phospho*enol*pyruvate carboxylase (*FtppcA1)* truncated proximal promoter constructs. Constructs are depicted in beige and segmented based on truncation sites. The coordinates of the truncation sites are shown above the promoter maps and are relative to the first nucleotide upstream of the *F. trinervia* PPCA1 translational start site (ATG), shown in green, and referred to as –1. Truncation sites correspond to the name of the fragments; *FtppcA1*(–570), *FtppcA1*(–543), *FtppcA1*(–472), and *FtppcA1*(–362). The proximal promoter is segmented into nucleotide regions (R1-4), defined by the truncation sites. The position of the predicted TATA box and transcriptional start site (TSS) are denoted by arrowheads above the constructs. **B** Activity of *F. trinervia ppcA1* promoter-reporter constructs in transiently transformed *F. bidentis* leaves. Mean ratios of firefly luciferase to *Renilla* luciferase are depicted as columns. All means are normalized to the mean of the *FtppcA1*(–570) construct. The normalized mean ratios of the technical triplicates for each transformed leaf disk are plotted in purple. Significance (*α* = 0.05) is denoted by letters above the data

**Fig. 7:**
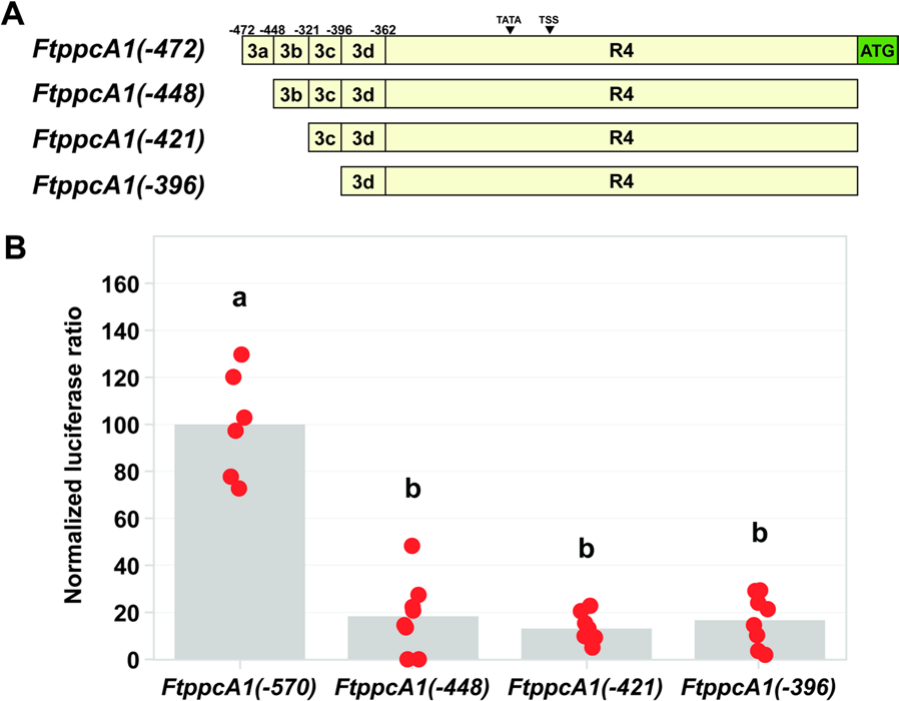
The promoter region controlling quantitative expression of *Flaveria trinervia* phospho*enol*pyruvate carboxylase. **A** Maps of *Flaveria trinervia* C_4_-associated phospho*enol*pyruvate carboxylase (*FtppcA1*) truncated proximal promoter constructs. Constructs are depicted in beige and segmented based on truncation sites. The coordinates of the truncation sites are shown above the maps and are relative to the first nucleotide upstream of the *F. trinervia* PPCA1 translational start site (ATG), shown in green, and referred to as –1. Truncation sites correspond to the name of the fragments; *FtppcA1*(–448), *FtppcA1*(–421), and *FtppcA1*(–396). The construct *FtppcA1*(–472) is described in Fig. 6 and is depicted here for clarity. The position of the predicted TATA box and transcriptional start site (TSS) are denoted by arrowheads above the constructs. **B** Activity of *F. trinervia ppcA1* promoter-reporter constructs in transiently transformed *F. bidentis* leaves. Mean ratios of firefly luciferase to *Renilla* luciferase are depicted as columns. All means are normalized to the mean of the *FtppcA1*(–570) construct (Fig. 6). The normalized mean ratios of the technical triplicates for each transformed leaf disk are plotted in orange. Significance (*α* = 0.05) is denoted by letters above the data

To determine the applicability of the *F. bidentis* leaf transient transformation system to the investigation of transcriptional regulation, a dual-luciferase binary vector containing a firefly and *Renilla* luciferase expression cassette was used. The transgene encoding *Renilla* luciferase (*Rluc*) was under the constitutive control of the *A. thaliana TCTP1* promoter, which enabled luminescence normalization and accounted for variations in transformation efficiency [56]. By contrast, the transgene encoding firefly luciferase (*Fluc*) was under the control of the experimental upstream regulatory regions; namely, truncated versions of the *FtppcA1* proximal promoter (Figs. 6 and 7).

Quantitative assays of firefly luciferase reporter gene expression in leaf extracts of *F. bidentis* plants transiently transformed with *FtppcA1* promoter-reporter constructs showed that the truncation constructs *FtppcA1(*−*543)::LUC* and *FtppcA1(*−*472)::LUC* exhibited no significant difference in luminescence ratio relative to the full-length *F. trinervia ppcA1* proximal promoter, *FtppcA1(–570)::LUC* (Fig. 6). However, transient transformation using the *FtppcA1(–362)::LUC* construct displayed a 3.19-fold reduction in relative luminescence, compared to *FtppcA1(*−*570)::LUC* (Fig. 6). This implicates a 110 bp region of the promoter, between nucleotides –472 and *–*362 (R3; Fig. 6A), in the quantitative regulation of *F. trinervia ppcA1*.

A second set of truncation constructs was created to interrogate the above 110 bp region (R3; Fig. 6A) of the *F. trinervia ppcA1* proximal promoter (Fig. 7). The three constructs *FtppcA1(*−*448)::LUC*, *FtppcA1(–421)::LUC*, and *FtppcA1(–396)::LUC*, returned luminescence ratios that were significantly less than the full-length proximal promoter, *FtppcA1(–570)::LUC* (Fig. 7B). Furthermore, the promoter activities of these three constructs were not significantly different to the activity of the *FtppcA1(*−*362)::LUC* construct (*cf.* Figures 6B and 7B). These results suggest that the upstream region responsible for quantitative expression of *F. trinervia ppcA1* lies in the 24 bp region of segment R3a, between nucleotides −472 and −448 (Fig. 7).

**Fig. 8:**
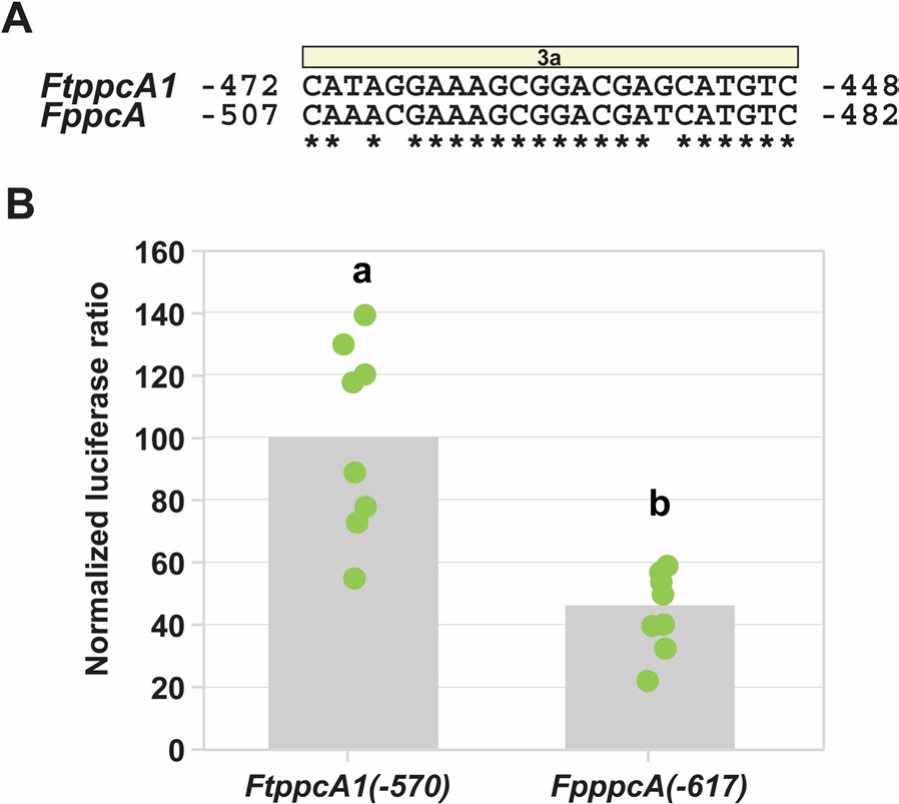
Comparison of phospho*enol*pyruvate carboxylase proximal promoters from *Flaveria trinervia* and *Flaveria pringlei*. **A** Nucleotide sequence alignment of the *Flaveria trinervia* C_4_-associated phospho*enol*pyruvate carboxylase (*FtppcA1*) proximal promoter region 3a to the homologous region of the *F. pringlei* ortholog (*FpppcA*). Asterisks indicate nucleotide conservation. Numerals indicate distance, in base pairs, from the translational start site. **B** Activity of *F. trinervia ppcA1* and *F. pringlei ppcA* promoter-reporter constructs in transiently transformed *F. bidentis* leaves. Mean ratios of firefly luciferase to *Renilla* luciferase are depicted as columns. All means are normalized to the mean of the *FtppcA1*(–570) construct (Fig. 6). The normalized mean ratios of the technical triplicates for each transformed leaf disk are plotted in green. Significance (*α* = 0.05) is denoted by letters above the data

To investigate the evolutionary relevance of the *F. tinervia ppcA1* results, the orthologous proximal promoter region of *ppcA* from the C_3_ congener *F. pringlei* was isolated. This region is 617 bp in length and displays 91% homology with *FtppcA1(*−*570)* (Supplementary Fig. S1) and exhibits three variant nucleotides in R3a (Fig. 8A). As shown in Fig. 8B, when used in our transient transformation-luciferase assay system, the C_4_ *F. trinervia* proximal promoter region (*FtppcA1(*−*570)::LUC*) exhibited a 2.5-fold higher relative luciferase activity than the orthologous region from *F. pringlei ppcA* (*FpppcA(*–*617)::LUC*).

## Discussion

The current study is the first report of an *Agrobacterium*-mediated transient transformation system for a C_4_ plant. The range of applications of this technology alongside its ease of use, speed, and capacity for high throughput have the potential to increase the understanding of the evolution of C_4_ photosynthesis in the genus *Flaveria*, as well as in other C_4_ lineages.

### Development of a transient transformation system for *Flaveria bidentis* leaves

The robust transient transformation system for intact *F. bidentis* leaves via an *Agrobacterium*-mediated approach reported in this study results in a transformation efficiency of 74.1% (Fig. 4), which is comparable to *Agrobacterium*-mediated transient transformation protocols developed for *N. benthamiana*, *A. thaliana*, and other species [48]. Both the basipetal developmental zone and acetosyringone concentration selected were critical for the high transformation efficiency observed. The upper-middle leaf zone exhibited the highest transformation efficiency relative to the proximal and distal zones (Fig. 3). The discrepancies in transient transformation efficiency observed for the different leaf zones may be due to the differences in cell organization and parenchymatous airspace volume along the length of the leaf. Transverse *F. bidentis* leaf sections display a reduced intercellular airspace volume in the proximal zone of the leaf relative to the upper middle and distal zones [16]. A similar trend was observed in poplar cultivars in which transient transformation efficiency was correlated with diffusion of infiltrate through the leaf lamina [61]. Consequently, this characteristic may influence the degree of infiltration and, by extension, the transient transformation efficiency of *F. bidentis* leaves. The age and distinct biochemical properties of cells along the leaf developmental gradient [16] may also confer different transformation efficiencies, thereby contributing to the observed *F. bidentis* leaf transient transformation pattern.

An increase in transformation efficiency was also found to be concomitant with an increase in acetosyringone concentration from 0.1 to 1 mM (Fig. 3). Acetosyringone is a phenolic phytohormone that induces *Agrobacterium* virulence [59, 62, 63], and its use in the *Agrobacterium* pre-culture has been shown to influence transient transformation efficiency in numerous species [61, 64, 65]. Our results are consistent with the proposed mechanism of acetosyringone action on both *Agrobacterium* and plant cells [59], and its effect on transient transformation efficiency [60]. In addition, the effectual concentration of the phytohormone in *F. bidentis* leaf transient transformation is comparable to those reported for other transient methods [61].

### *Flaveria bidentis* co-transformation and its applications

The above discussion of transformation efficiency is based on experiments of *F. bidentis* leaves transformed with *Agrobacterium* suspensions containing a single reporter gene construct; however, transient transformation using a suspension of two *Agrobacterium* strains, each harboring a different plasmid with a distinct reporter gene, is also possible (see Supplementary Fig. S2). This extension of the technique demonstrates its breadth and flexibility, as many plant functional assays, such as bimolecular fluorescence complementation, Förster resonance energy transfer, trans-activation, and CRISPR-based assays, require two independent vectors [66–69]. The ability to transiently transform *F. bidentis* leaves with multiple vectors is, therefore, highly advantageous. However, wherever possible, it is preferable to consolidate the transgenes into a single vector, thereby avoiding the occurrence of numerous singly transformed cells. For example, comparisons of the GFP, red fluorescent protein (RFP), and merged GFP/RFP images shown in Supplementary Fig. S2 indicate that not all transformed cells express both GFP and RFP.

### Transformability of *Flaveria bidentis* leaf cell types

To investigate the transformability of *F. bidentis* leaf bundle-sheath cells, a *FbRbc*S1-*gfp* construct designed by Patel et al. [54], which confers bundle-sheath cell-specific expression, was used. No transiently transformed cells were observed in the *F. bidentis* leaves infiltrated with this construct (Fig. 5). This is in contrast to constitutive and mesophyll cell-specific promoter-reporter constructs, which resulted in numerous transformed cells (Figs. 3, 4, 5; Supplementary Figs S2 and S3). Transformed elongate cells flanking the vasculature were detected in experiments using constitutively expressed reporter genes (Supplementary Fig. S3), though no consistent bundle-sheath cell labelling was observed. The precise mechanism by which *Agrobacterium* T-DNA enters a plant cell is unknown. Though various mechanisms have been proposed [70], all are contingent on the proximity of *Agrobacterium* cells to plant cells; therefore, transformation is presumed to be dependent upon plant cell accessibility. *F. bidentis* leaves exhibit Kranz anatomy, in which mesophyll cells are concentrically arranged around bundle-sheath cells; accordingly, few bundle-sheath cells are adjacent to intracellular air spaces [13]. Consequently, bundle-sheath cell accessibility is limited and this characteritstic may explain the lack of transformed bundle-sheath cells in the transient assays.

### A 24 bp region is responsible for the quantitative expression of *ppcA1* in *Flaveria trinervia*

To illustrate the further utility of the newly developed *F. bidentis* leaf transient transformation protocol, a dual-luciferase reporter assay was used to assess the activity of *FtppcA1* promoter-reporter constructs. The *ppcA1* gene encoding the C_4_-associated PEPC in *F. trinervia* exhibits high levels of mesophyll cell-specific expression. Though the MEM1 element in the distal promoter region is responsible for the cell-type expression pattern, an unknown element (or elements) in the proximal promoter was predicted to be responsible for quantitative expression [22]. In the current study, two sets of promoter truncations were used to refine the promoter region controlling high levels of *ppcA1* expression. The first set of promoter truncation constructs implicated a 110 bp region between −472 and −362 in the quantitative activity of the *F. trinervia ppcA1* promoter (Fig. 6B). A second set of truncation constructs further refined the region responsible for the high expression of *ppcA1 in F. trinervia* (Fig. 7). Each of these promoter truncations exhibited reduced activity relative to the full-length proximal promoter, and equivalent activity to the *FtppcA1(*−*362)* construct (*cf.* Figures 6 and 7). Consequently, the deletion of R1 to R3a (nucleotides −570 to −448; Figs. 6 and 7) was determined to be the minimum truncation required to abolish the high activity of the full-length *FtppcA1* proximal promoter. Accordingly, the deletion of R1-R2 (nucleotides −570 to −472; Fig. 6) was determined to be the maximum truncation that retained the promoter activity of the 570 bp proximal promoter. Taken together, these results indicate the 24 bp region referred to as R3a, between −472 and −448 (Fig. 7A), is key for the quantitative expression of *F. trinervia ppcA1* (Fig. 7B). These results support the earlier hypothesis of a CRE (or CREs) in the *F. trinervia ppcA1* proximal promoter region being responsible for high levels of *ppcA1* expression and working synergistically with the distal MEM1 enhancer, which drives mesophyll cell-specific expression [22, 24].

Comparison of the C_4_ *F. trinervia ppcA1* and C_3_ *F. pringlei ppcA* proximal promoters illustrated a 2.5-fold greater relative luciferase activity for the *F. trinervia* proximal promoter than the *F. pringlei* region in *F. bidentis* leaves (Fig. 8B). This relationship further supports the hypothesis that a CRE, present in the C_4_ proximal promoter, but absent in the C_3_ proximal promoter, confers quantitative expression of C_4_ *ppcA1*. The *F. trinervia* promoter truncation analysis identified a 24 bp region where this putative CRE may be located (Fig. 7). An alignment of the *F. trinervia* and *F. pringlei* proximal promoters reveals a high degree of conservation in this 24 bp region, R3a, with the exception of three substitutions (Fig. 8A; Supplementary Fig. S1). Namely, an A-to-T transversion at position −470, a C-to-G transversion at position −468, and a T-to-G transversion at position −455, relative to the *F. trinervia ppcA1* translational start site (Fig. 8A). Further promoter-reporter experiments may elucidate which, if any, of these substitutions is/are responsible for the difference in proximal promoter activities of *F. trinervia ppcA1* and *F. pringlei ppcA* and, therefore, the mutation that enables high *ppcA1* expression in C_4_ *Flaveria* species.

The quality of the data reported here (Figs. 6, 7, 8) is comparable to data generated in other species using similar quantitative transient transformation approaches; for example, the dual-luciferase system used in *A. thaliana* protoplast transfection experiments reported by Lloyd et al. [55]. For each upstream region tested in the current study, data with low standard error values were generated (Supplementary Table S2). This shows that the results of this novel *F. bidentis* leaf transformation system are reliable and consistent, both within single leaves on individual plants as well as across leaves from different individuals (Figs. 6B, 7B, and 8B). Furthermore, the consistency of the results from each transformation experiment is testament to the reproducibility of this workflow. This precision and replicability resulted in few data points being required to determine statistical significance (Figs. 6B, 7B, and 8B), which enables a rapid experimental timeframe in which a statistically sufficient sample size can be generated (*i.e.,* two successive experiments within two weeks). This time frame is in stark contrast to the stable transformation protocol developed by Chitty et al. [18], which has previously been used to explore gene regulation in *F. bidentis* [24], but can take more than 20 weeks before reporter gene assays can be carried out. The results presented here describe a technology that allows the rapid investigation of transcriptional regulation of genes encoding key C_4_ cycle enzymes in the genus *Flaveria* which may be used alone or to complement a stable transformation approach.

### The *Flaveria bidentis* transient transformation system as a platform for the development of other transformation systems

The establishment of the *F. bidentis* leaf transient transformation protocol means that genetic elements from *Flaveria* congeners can be assayed in an intrageneric transient expression system. This is a significant advancement from the use of *N. benthamiana*, which represents an interfamilial transient transformation system. However, it also introduces the possibility of developing a similar system for other *Flaveria* species, for example a C_3_ species. This would allow comparisons of CREs in different *trans*-regulatory contexts. Additionally, the development of this protocol may have repercussions on the stable transformation of *F. bidentis* beyond being a tool for preliminary confirmation of transgene expression. The leaf disk regeneration-based stable transformation of *N. benthamiana* [71] in combination with the *F. bidentis* protocol described here may serve as a starting point for the development of an alternative *F. bidentis* stable transformation system and indeed, the stable transformation of other *Flaveria* species, contingent on their transient transformation, as has been the case for other plant species [72, 73].

## Conclusions

A robust and efficient protocol for the transient transformation of *F. bidentis* leaves has been presented. For many studies of gene function and C_4_ evolution, this protocol has distinct temporal, logistical, and technical advantages over the pre-existing *F. bidentis* stable transformation system [18]. Furthermore, this protocol provides a distinct phylogenetic advantage over similar agroinfiltration techniques to the investigation of *F. bidentis* genetic elements by allowing them to be investigated in a homologous or intrageneric context, as in the case of the *ppcA1* proximal promoter of *F. trinervia*. Collectively, the *Agrobacterium*-mediated transient transformation of *F. bidentis* leaves will aid in the understanding of the evolution of C_4_ photosynthesis, both in the genus *Flaveria*, and in other C_4_ lineages.

## Supplementary Information

Supplementary material 1: Table S1. Sequences of primers used in this study. *Xba*I and *Nco*I recognition sites shown in bold. Table S2. Mean and standard error values of luciferase quantification experiments. Figure S1. Nucleotide sequence alignment of the phospho*enol*pyruvate carboxylase proximal promoter regions of *Flaveria* *pringlei* and *F. trinervia*. Figure S2. Capacity of the *Flaveria* *bidentis* leaf transient transformation system for visualizing multiple reporter constructs. Figure S3. The transformability of bundle-sheath cells using the *Flaveria* *bidentis* leaf transient transformation system

## Data Availability

All data generated or analyzed during this study are included in this published article [and its supplementary information files].
