# Supplementary material for: Agrobacterium-mediated transient transformation of Flaveria bidentis leaves: a novel method to examine the evolution of C4 photosynthesis
Source: Plant Methods. 2024 Dec 27;20:193. doi: 10.1186/s13007-024-01306-z (PMC11674322; doi:10.1186/s13007-024-01306-z)
Supplement: Supplementary file 1 — Supplementary material 1: Table S1. Sequences of primers used in this study. XbaI and NcoI recognition sites shown in bold. Table S2. Mean and standard error values of luciferase quantification experiments. Figure S1. Nucleotide sequence alignment of the phosphoenolpyruvate carboxylase proximal promoter regions of Flaveria pringlei and F. trinervia. Figure S2. Capacity of the Flaveria bidentis leaf transient transformation system for visualizing multiple reporter constructs. Figure S3. The transformability of bundle-sheath cells using the Flaveria bidentis leaf transient transformation system [file 13007_2024_1306_MOESM1_ESM.docx]

ADDITIONAL FILE 1

**Supplementary Table S1. Sequences of primers used in this study. *Xba*I and *Nco*I recognition sites shown in bold.**

| **Name** | **Sequence 5′ to 3′** |
| --- | --- |
| *FtppcA1* upstream region (FWD) | **TCTAGA**GCTTATGTTTGTTGGTAG |
| *FtppcA1* upstream region (REV) | **CCATGG**CTCACACCCTTGCTTAATAC |
| *FtppcA1(****–****570)* proximal promoter (FWD) | **TCTAGA**CGGTGTTAATGATGGATGATG |
| *FtppcA1(****–****543)* proximal promoter (FWD) | **TCTAGA**GACATCGTTTTAATACTAATTG |
| *FtppcA1(****–****472)* proximal promoter (FWD) | **TCTAGA**CATAGGAAAGCGGACGA |
| *FtppcA1(****–****362)* proximal promoter (FWD) | **TCTAGA**GAATTCTTCAATCCTGAGTTTGC |
| *FpppcA(****–****617)* proximal promoter (FWD) | **TCTAGA**CGTTTCTAAATAAATTATATTTAAAAACATGCC |
| *FpppcA(****–****617)* proximal promoter (REV) | **CCATGG**CACACACACTTGCTTAATTCC |

**Supplementary Table S2. Mean and standard error values of luciferase quantification experiments.**

|  | **Construct** | **Mean** | **Standard Error** |
| --- | --- | --- | --- |
| **Figure 6** | *FtppcA1(–570)::LUC* | 100.00 | 7.57 |
|  | *FtppcA1(–543)::LUC* | 97.01 | 9.84 |
|  | *FtppcA1(–472)::LUC* | 117.24 | 4.20 |
|  | *FtppcA1(–362)::LUC* | 22.85 | 5.49 |
| **Figure 7** | *FtppcA1(–570)::LUC* | 100.00 | 8.40 |
|  | *FtppcA1(–448)::LUC* | 18.33 | 4.10 |
|  | *FtppcA1(–421)::LUC* | 13.11 | 2.49 |
|  | *FtppcA1(–396)::LUC* | 16.72 | 4.15 |
| **Figure 8** | *FtppcA1(–570)::LUC* | 100.00 | 10.79 |
|  | *FpppcA(–617)::LUC* | 46.15 | 4.56 |


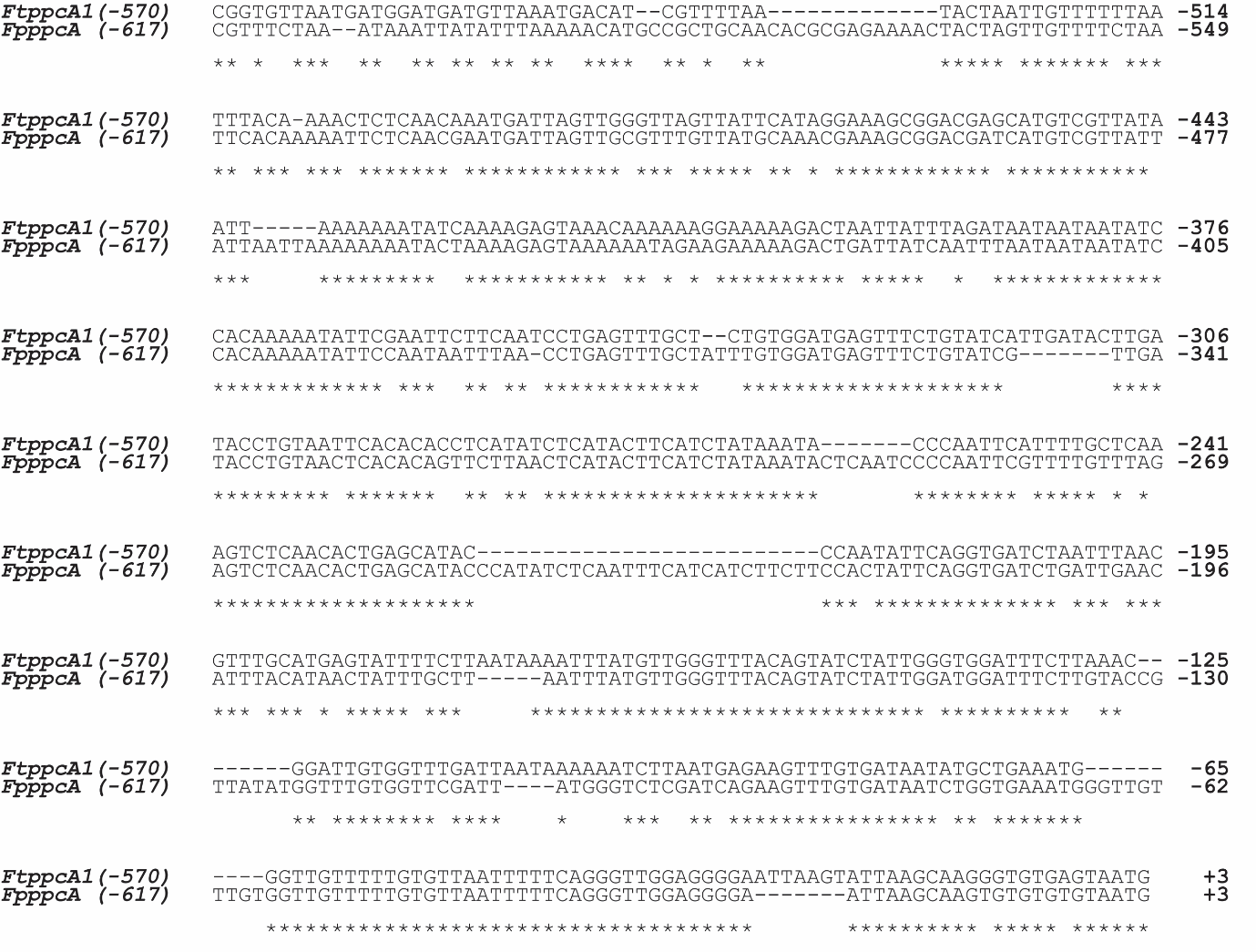


**Supplementary Figure S1.** **Nucleotide sequence alignment of the phospho*enol*pyruvate carboxylase proximal promoter regions of *Flaveria pringlei* (C_3_) and *F. trinervia* (C_4_).** The *F. trinervia ppcA1* proximal promoter is denoted as *FtppcA1(–570)* and the *F. pringlei ppcA* proximal promoter is denoted as *FpppcA(****–****617)*. Asterisks denote conservation across the two sequences. Numerals to the right of the nucleotide sequence alignment indicate distance, in base pairs, from the translational start site (ATG).


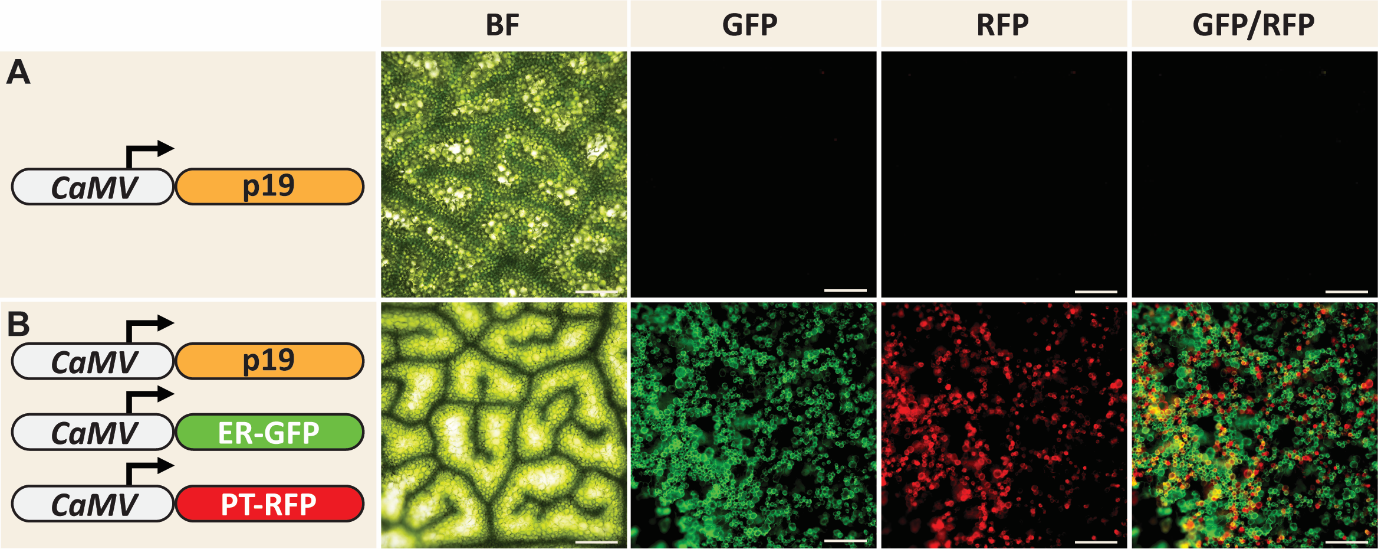


Supplementary Figure S2. Capacity of the *Flaveria bidentis* leaf transient transformation system for visualizing multiple reporter constructs. (A) Leaves were infiltrated with an *Agrobacterium* suspension harboring a plasmid containing the gene encoding p19 under the control of the cauliflower mosaic virus 35S promoter (CaMV) as a negative control. (B) Leaves were infiltrated with a co-suspension of *Agrobacterium* strains independently containing genes encoding the p19 protein, an endoplasmic reticulum-targeted green fluorescent protein (ER-GFP), or a plastid-targeted red fluorescent protein (PT-RFP; [58]), all under the control of the CaMV promoter. Leaves were visualized three days post-infiltration via brightfield (BF) and epifluorescence microscopy with a GFP filter set (excitation; 454-490 nm, emission collection; 500-540 nm) and an RFP filter set (excitation; 550-590 nm, emission collection; 608-683 nm). Co-transformed cells are observed in the merged GFP/RFP channel and display as yellow. Scale bar = 200 μm.


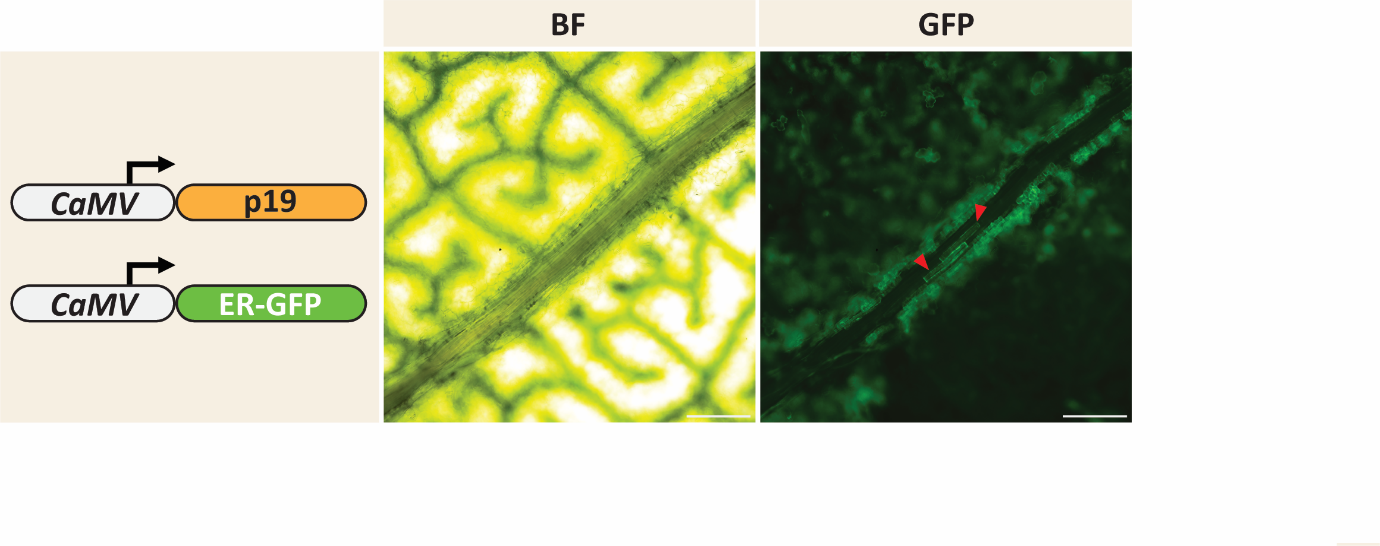


**Supplementary Figure S3. The transformability of bundle-sheath cells using the *Flaveria bidentis* leaf transient transformation system.** A *Flaveria bidentis* leaf was infiltrated with a co-suspension of *Agrobacterium* strains independently harboring vectors containing genes encoding the p19 protein or an endoplasmic reticulum-targeted green fluorescent protein (ER-GFP), both under the control of the cauliflower mosaic virus 35S (CaMV) promoter [58]. Red arrowheads indicate transformed bundle-sheath cells. Leaves were visualized three days post-infiltration via brightfield (BF) and epifluorescence microscopy with a GFP filter set (excitation; 454-490 nm, emission collection; 500-540 nm). Scale bar = 200 μm.
